# A longitudinal study of men with male genital schistosomiasis in southern Malawi associated with human, zoonotic and hybrid schistosomes

**DOI:** 10.1017/S0031182025100942

**Published:** 2025-12

**Authors:** Bright Mainga, Sekeleghe Kayuni, Fatima Ahmed, Guilleary Deles, Lucas Joseph Cunningham, Dingase Kumwenda, David Lally Jnr, Priscilla Chammudzi, Donales Kapira, Gladys Namacha, Alice Chisale, Tereza Nchembe, Louis Kinley, Ephraim Chibwana, Bazwell Nkhalema, Gilbert Chapweteka, Henry Chibowa, Victor Kumfunda, Alexandra Juhasz, Sam Jones, John Archer, Angus M O'Ferrall, Sarah Rollason, Abigail Cawley, Ruth Cowlishaw, Andrew Nguluwe, John Chiphwanya, Michael Luhanga, Holystone Kafanikhale, Peter Makaula, E. James La Course, Janelisa Musaya, J. Russell Stothard

**Affiliations:** 1 Malawi Liverpool Wellcome Programme, Kamuzu University of Health Sciences, Queen Elizabeth Central Hospital Campus, Chichiri, Blantyre 3, Malawi; 2 Laboratory Department, Mangochi District Hospital, Mangochi, Malawi; 3 Department of Tropical Disease Biology, Liverpool School of Tropical Medicine, CTID Building, Liverpool, Merseyside, UK; 4 Pathology Department, School of Medicine and Oral Health, Mahatma Gandhi Campus, Chichiri, Blantyre 3, Malawi; 5 Obstetrics and Gynaecology Department, Queen Elizabeth Central Hospital, Blantyre, Malawi; 6 Radiology Department, Queen Elizabeth Central Hospital, Blantyre, Malawi; 7 Nsanje District Hospital, Ministry of Health, Nsanje, Malawi; 8 Mangochi District Hospital, Ministry of Health, Mangochi, Malawi; 9 Institute of Medical Microbiology, Semmelweis University, Budapest, Hungary; 10 School of Biosciences, The Sir Martin Evans Building, Cardiff University, Cardiff, UK; 11 National Schistosomiasis and Soil-Transmitted Helminths Control Programme, Community Health Sciences Unit (CHSU), Ministry of Health, Lilongwe, Malawi

**Keywords:** hybrid, Lake Malawi, MGS, praziquantel, *Schistosoma haematobium*, *Schistosoma mattheei*, semen, Shire river

## Abstract

In sub-Saharan Africa’s endemic areas for urogenital schistosomiasis, male genital schistosomiasis (MGS) can cause significant morbidity. As part of the *Hybridization in UroGenital Schistosomiasis* investigation, an MGS sub-study examined a cohort of adult men over a calendar year to better ascertain general infection dynamics and putative zoonotic schistosome transmission. During follow-up, demographic, health and socio-economic data were collected through individual questionnaire interviews. Collected urine and semen were analysed using urine filtration, urine and semen microscopy and molecular DNA analyses of semen. Ten participants with reported MGS-associated symptoms had *Schistosoma* eggs in their urine and semen at 6-month follow-up, with seven at 12 months. Ten out of 11 participants with *Schistosoma haematobium* eggs on semen microscopy at baseline had persistent infection at 6-month follow-up, together with 6 new participants, giving an MGS prevalence of 84·2% (*n* = 19). Two also had *Schistosoma mattheei* eggs co-infection. Four of the 13 participants at 12-month follow-up had *S. haematobium* eggs in their semen which were persistent at all the time points. Using semen PCR, 14 participants (73·7%) had *Schistosoma* infection at 6 months, with only 2 participants being infected for first time. Upon DNA analysis, three participants also had hybrid co-infection at this time point. At 12 months, only 6 participants had *Schistosoma* infection with no hybrids detected. In summary, like *S. haematobium* and despite praziquantel treatment, both zoonotic and hybrid schistosomes can continue to cause MGS, which pose a further tangible challenge in future management and control measures.

## Graphical abstract

**Figure:**
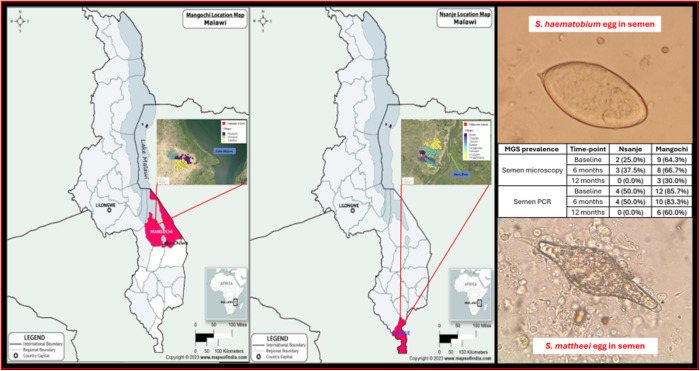

## Introduction

The WHO published a road map for control and elimination of neglected tropical diseases (NTDs) 2021–2030 of which one of their public health targets is specific for schistosomiasis (WHO, 2022). Despite being targeted for elimination as a public health problem, schistosomiasis is one of the NTDs that continue to affect millions of people, particularly in resource-limited regions of the world (McManus et al. 2018; Buonfrate et al. 2025). Despite being a major public health issue in many tropical and subtropical countries, schistosomiasis has often been overlooked in global health discussions and funding priorities. In sub-Saharan Africa (SSA), male genital schistosomiasis (MGS), a gender-specific manifestation of urogenital schistosomiasis in men, has a long history of being an under-recognized and under-reported largely due to limited awareness and access to diagnostic tools, particularly resource poor rural areas where urogenital schistosomiasis is endemic (Bustinduy et al. 2022). This is in contrast with female genital schistosomiasis (FGS) which has been widely studied, diagnostic, management and control strategies developed and advocated (WHO, 2015).

The condition, MGS, was first described in 1911 (Madden, 1911), though it likely existed much earlier in SSA and the Middle East regions, where *Schistosoma haematobium* was prevalent (Kayuni et al. 2019a). Men infected after exposure to schistosome cercariae-infested water bodies were observed to have genital lesions, including fibrosis in scrotum, prostate and seminal vesicles, testicular atrophy, and even infertility due to inflammatory response to tissue eggs deposition (Costain et al. 2018).

In recent years, it has been demonstrated that *S. haematobium* has an innate capacity to form viable hybrids with other schistosome species, particularly with *Schistosoma mattheei*, a common species of animal schistosome found in livestock (Stothard et al. 2020, 2024). Hybridization is a well-established evolutionary mechanism that contributes to genetic diversity, allowing species to adapt more quickly to environmental changes (Mawa et al. 2021). In light of the future challenge to interrupt schistosome transmission, there is growing interest in hybrid schistosomes, and their environmental dynamics, resulting from crossbreeding between zoonotic and human *Schistosoma* species. Furthermore, such introgressed or hybrid variants can potentially complicate the clinical picture of urogenital schistosomiasis and how the parasite is evolving and adapting to different hosts (Rey et al. 2021; Kayuni et al. 2024b).

Certainly, zoonotic schistosomes have contributed significantly to the overall burden of schistosomiasis in humans, with some causing fibrosis and calcification in prostate, seminal vesicles and testes (Buonfrate et al. 2025; Léger et al. 2020). Advancements in molecular biology significantly improved modern diagnostic tools for schistosomiasis, including MGS where detection of *Schistosoma* eggs, DNA and antigens can be made directly from semen, prostate biopsies, and other tissue samples, offering more precise and reliable diagnosis (Cunningham et al. 2024, Rinaldi et al. 2015).

Following a baseline MGS survey within the longitudinal study entitled *Hybridization of UroGenital Schistosomiasis* (HUGS) in Mangochi and Nsanje districts, Southern Malawi (Kayuni et al. 2024a), a clinical 12-month follow-up was conducted on participating men to describe progression of schistosome infections either from human, zoonotic and hybrid worms undertaken between June 2023 and July 2024.

## Materials and methods

### Study sites

**Figure 1.**
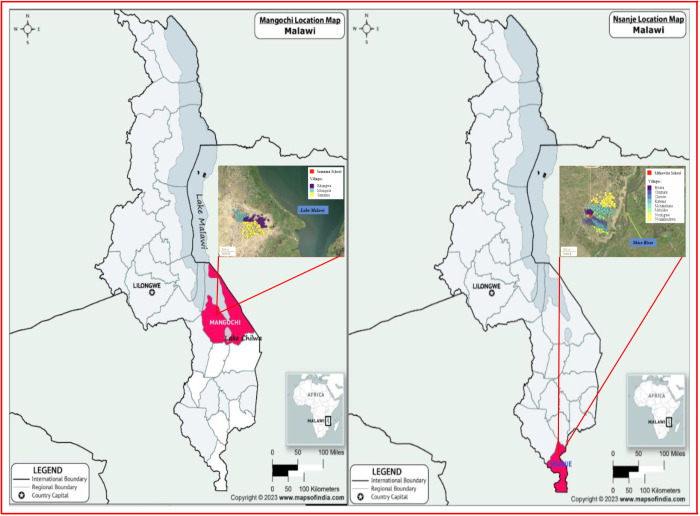
Map showing two study communities around Samama School in Mangochi District and Mthawira School in Nsanje District of Southern Malawi where participates came from.

This MGS longitudinal study was conducted in the two sentinel communities at Samama and at Mthawira in Mangochi and Nsanje Districts, respectively (Figure 1). Here, hybrid infections have previously been identified in school children in these communities, with a tenth of infected children shedding atypical eggs in urine, alongside ectopic egg patent *S. mansoni* and zoonotic eggs, at the baseline of the HUGS study in 2022.

### Study population

The study population comprised of all males aged between 18 and 45 years old who had active egg-patent schistosomiasis at baseline in 2022 in the two study sites of Samama and Mthawira areas. These were recruited as at baseline of the MGS study in June and July 2023 and underwent a 6- and 12-month follow-up. Written informed consent was sought after adequate awareness before enrolment into the study. All participants were provided a directly observed treatment with praziquantel standard dose, 40 mg/kg body weight, at the end of each study visit.

### Data collection

Demographic data were gathered through individual questionnaire interviews to collect details on participants’ health, socio-economic status, water contact habits, livestock availability and symptoms associated with MGS (Kayuni et al. 2024a).

### Urine and semen microscopy

A 120 mL clear container was used to collect a urine sample during the study visit, which was then filtered and examined microscopically to detect *Schistosoma* eggs in 10 mL of the well-mixed sample (Cheesbrough, 2005). Additionally, each sample underwent reagent dipstick testing and point-of-care circulating cathodic antigen (POC-CCA) analysis for detection of intestinal schistosomiasis. Participants were then requested to provide a semen sample, which was collected in a clear plastic bag for direct field microscopy (Kayuni et al. 2019b). The samples were subsequently centrifuged, and the sediments were re-examined microscopically. These sediments were preserved in 1 mL of 70% ethanol to prepare for shipment to Liverpool School of Tropical Medicine (LSTM) in the United Kingdom.

### Molecular analyses

As previously described (Cunningham et al. 2024; Kayuni et al. 2024a), molecular analysis of the semen sediments included High-Resolution Melt (HRM) and TaqMan® real-time PCR for *Schistosoma* spp. DNA markers for viral infection with HPV were also screened for using the QIAscreen HPV PCR Test kit (Qiagen, Manchester, UK), capable of screening for two high-risk genotypes, 16 and 18 alongside the others. Also, seminal sediments were analysed for other STIs such as *Chlamydia trachomatis, Trichomonas vaginalis, Herpes simplex* virus (HSV) 1, HSV 2, *Candida* spp., *Ureaplasma* spp., *M. hominis, S. agalactiae* using the EasyScreen™ STI Kit (SydPath, Sydney, Australia).

### Data analyses

The results of the diagnostic tests were analysed using non-parametric tests due to the small sample size and non-normal distribution. These were tabulated and presented accordingly.

### Ethical considerations

Ethical approval for the study was granted by the College of Medicine Research Ethics Committee (COMREC), Kamuzu University of Health Sciences (KUHeS), Malawi, (Approval number: P.08/21/3381) and the LSTM Research Ethics Committee (LSTM REC) in the United Kingdom (registration number: 22-028). Informed consent to participate in the study was obtained from all of the participants. Privacy and confidentiality were maintained throughout the study. All participants including those who had *Schistosoma* eggs in urine and/or semen were provided a directly observed treatment with praziquantel standard dose, 40 mg/kg body weight, at each time point. Appropriate referral for treatment and management was provided to participants who had other infections.

## Results

Of the 22 male participants recruited into the MGS sub-study at baseline in June 2023, 19 (7 in Nsanje, 12 in Mangochi) and 14 (3 in Nsanje, 11 in Mangochi) participants were available for follow-up at 6- and 12-month time points, respectively (Table 1).

**Table 1.** Demographical information and laboratory findings of the participants at all the time points

ID	Age (years)	Urine filtration egg count			Semen microscopy						Observations on semen microscopy		
		Baseline	6 months	12 months	Baseline		6 months		12 months		Baseline	6 months	12 months
					*S. h.*	*S. matt.*	*S. h.*	*S. matt.*	*S. h.*	*S. matt.*			
					eggs	eggs	eggs	eggs	eggs	eggs			
A	19	1−9	0	0	8	1	9	0	2 (cal)	0	Normal	Normal	Normal
B	18	50+	0	0	25	0	5	0	0	0	Normal	Normal	Normal
C	18	10−49	1−9	0	6	0	8	0	0	0	Haemospermia (blood in semen), loose consistency, azoospermia	Loose consistency, azoospermia	Loose consistency, azoospermia
D	18	0	0	0	15	0	10	0	1 (cal)	0	Colour change, loose consistency, azoospermia	Normal	Normal
E	21	0	1−9	0	2	0	3	0	0	0	Normal	Few live spermatozoa	Some fragmented spermatozoa
F	28	0	0	0	0	0	6	0	0	0	Normal	Normal	Normal
G	27	1−9	0	0	1	0	8	2	2	1	Normal	Dead spermatozoa	Dead spermatozoa
H	19	0	N/A	N/A	5	0	N/A	N/A	N/A	N/A	Normal	N/A	N/A
I	30	1−9	10−49	0	0	0	0	0	0	0	Normal	Normal	Normal
J	22	10−49	0	50+	9	0	7	0	N/A	N/A	Normal	Normal	Normal
K	18	0	N/A	N/A	0	0	N/A	N/A	N/A	N/A	Normal	N/A	N/A
L	21	0	1−9	1−9	0	0	4	0	0	0	Normal	Dead spermatozoa	1/3 dead spermatozoa
M	18	10−49	50+	10−49	100	0	8	1	4	0	Haemospermia, loose consistency, dead spermatozoa	Azoospermia	Loose consistency, azoospermia
N	32	1−9	50+	N/A	0	0	7	0	N/A	N/A	Normal	Normal	N/A
O	32	0	N/A	N/A	0	0	N/A	N/A	N/A	N/A	Normal	Normal	Normal
P	39	0	0	1−9	0	0	10	0	0	0	Normal	Normal	Normal
Q	30	0	0	0	0	0	0	0	0	0	Normal	Normal	Azoospermia
R	26	0	10−49	N/A	0	0	7	0	N/A	N/A	Normal	Normal	N/A
S	19	0	0	0	1	0	3	0	0	0	Normal	Cloudy, loose consistency	Cloudy, loose consistency, azoospermia
T	22	0	10−49	N/A	0	0	0	0	N/A	N/A	Normal	Normal	N/A
U	22	1−9	1−9	N/A	500	0	10	0	N/A	N/A	Normal	Normal	N/A
V	25	1−9	0	N/A	0	0	8	0	N/A	N/A	Normal	Normal	N/A

Note: *S. h. = Schistosoma haematobium, S. matt.* = *Schistosoma mattheei*, N/A = not available.

Three participants (J, L and O) reported symptoms associated with MGS at baseline, namely blood in semen (haemospermia), sores and pain in the genitalia, pain during coitus and ejaculation (Table 2). Only one participant (J) with symptoms had *Schistosoma* eggs in their urine and semen. At 6-month follow-up, 10 participants reported MGS symptoms while 7 participants reported the symptoms at 12-month follow-up time point.

**Table 2.** Symptoms of MGS experienced by the participants at all the time points

		Symptoms	A	B	C	D	E	F	G	H	I	J	K	L	M	N	O	P	Q	R	S	T	U	V	N in cohort
**Time point 1 – June 2023**	**Urinary**	Fever	1	1	1	0	0	0	1	0	1	1	0	0	1	1	0	0	0	0	0	0	1	1	**9**
		Headache	1	1	1	0	0	0	1	0	1	1	0	0	1	1	0	0	0	0	0	0	1	1	**9**
		Fatigue	1	1	1	0	0	0	1	0	1	1	0	0	1	1	0	0	0	0	0	0	1	1	**8**
		Abdominal pain	1	1	1	0	0	0	1	0	1	1	0	0	1	1	0	0	0	0	0	0	1	1	**5**
		Dysuria	1	1	1	0	0	0	1	0	1	1	0	0	1	1	0	0	0	0	0	0	1	1	**7**
		Polyuria	1	1	1	0	0	0	1	0	1	1	0	0	1	1	0	0	0	0	0	0	1	1	**10**
		Discoloured Urine	1	1	1	0	0	0	1	0	1	1	0	0	1	1	0	0	0	0	0	0	1	1	**8**
		Haematuria	1	1	1	0	0	0	1	0	1	1	0	0	1	1	0	0	0	0	0	0	1	1	**6**
		Blood in stool	1	1	1	0	0	0	1	0	1	1	0	0	1	1	0	0	0	0	0	0	1	1	**2**
	**Genital**	Haemospermia	1	1	1	1	1	0	1	1	0	1	0	0	1	0	0	0	0	0	1	0	1	0	**1**
		Dyspareunia	1	1	1	1	1	0	1	1	0	1	0	0	1	0	0	0	0	0	1	0	1	0	**2**
		Dysorgasmia	1	1	1	1	1	0	1	1	0	1	0	0	1	0	0	0	0	0	1	0	1	0	**2**
		Genital pain	1	1	1	1	1	0	1	1	0	1	0	0	1	0	0	0	0	0	1	0	1	0	**2**
		Genital sores	1	1	1	1	1	0	1	1	0	1	0	0	1	0	0	0	0	0	1	0	1	0	**3**
		**N sx experienced**	**0**	**3**	**0**	**0**	**6**	**4**	**1**	**0**	**1**	**13**	**0**	**6**	**6**	**2**	**11**	**2**	**3**	**7**	**1**	**4**	**1**	**3**	
**Time point 2 – January 2024**	**Urinary**	Fever	0	0	1	0	1	0	0	N/A	1	0	N/A	1	1	1	N/A	0	0	1	0	0	1	0	**12**
		Headache	0	0	1	0	1	0	0	N/A	1	0	N/A	1	1	1	N/A	0	0	1	0	0	1	0	**17**
		Fatigue	0	0	1	0	1	0	0	N/A	1	0	N/A	1	1	1	N/A	0	0	1	0	0	1	0	**11**
		Abdominal pain	0	0	1	0	1	0	0	N/A	1	0	N/A	1	1	1	N/A	0	0	1	0	0	1	0	**16**
		Dysuria	0	0	1	0	1	0	0	N/A	1	0	N/A	1	1	1	N/A	0	0	1	0	0	1	0	**12**
		Polyuria	0	0	1	0	1	0	0	N/A	1	0	N/A	1	1	1	N/A	0	0	1	0	0	1	0	**13**
		Discoloured Urine	0	0	1	0	1	0	0	N/A	1	0	N/A	1	1	1	N/A	0	0	1	0	0	1	0	**15**
		Haematuria	0	0	1	0	1	0	0	N/A	1	0	N/A	1	1	1	N/A	0	0	1	0	0	1	0	**11**
		Blood in stool	0	0	1	0	1	0	0	N/A	1	0	N/A	1	1	1	N/A	0	0	1	0	0	1	0	**5**
	**Genital**	Haemospermia	1	1	1	1	1	1	1	N/A	0	1	N/A	1	1	1	N/A	1	0	1	1	0	1	1	**3**
		Dyspareunia	1	1	1	1	1	1	1	N/A	0	1	N/A	1	1	1	N/A	1	0	1	1	0	1	1	**3**
		Dysorgasmia	1	1	1	1	1	1	1	N/A	0	1	N/A	1	1	1	N/A	1	0	1	1	0	1	1	**5**
		Genital pain	1	1	1	1	1	1	1	N/A	0	1	N/A	1	1	1	N/A	1	0	1	1	0	1	1	**7**
		Genital sores	1	1	1	1	1	1	1	N/A	0	1	N/A	1	1	1	N/A	1	0	1	1	0	1	1	**3**
		**N sx experienced**	**4**	**12**	**7**	**0**	**4**	**8**	**8**	**0**	**3**	**7**	**0**	**11**	**10**	**5**	**0**	**5**	**10**	**12**	**6**	**5**	**3**	**8**	
**Time point 3 – June 2024**	**Urinary**	Fever	0	0	0	0	0	0	0	N/A	0	1	N/A	1	1	N/A	N/A	1	0	N/A	0	N/A	N/A	N/A	**9**
		Headache	0	0	0	0	0	0	0	N/A	0	1	N/A	1	1	N/A	N/A	1	0	N/A	0	N/A	N/A	N/A	**12**
		Fatigue	0	0	0	0	0	0	0	N/A	0	1	N/A	1	1	N/A	N/A	1	0	N/A	0	N/A	N/A	N/A	**6**
		Abdominal pain	0	0	0	0	0	0	0	N/A	0	1	N/A	1	1	N/A	N/A	1	0	N/A	0	N/A	N/A	N/A	**8**
		Dysuria	0	0	0	0	0	0	0	N/A	0	1	N/A	1	1	N/A	N/A	1	0	N/A	0	N/A	N/A	N/A	**7**
		Polyuria	0	0	0	0	0	0	0	N/A	0	1	N/A	1	1	N/A	N/A	1	0	N/A	0	N/A	N/A	N/A	**8**
		Discoloured Urine	0	0	0	0	0	0	0	N/A	0	1	N/A	1	1	N/A	N/A	1	0	N/A	0	N/A	N/A	N/A	**8**
		Haematuria	0	0	0	0	0	0	0	N/A	0	1	N/A	1	1	N/A	N/A	1	0	N/A	0	N/A	N/A	N/A	**7**
		Blood in stool	0	0	0	0	0	0	0	N/A	0	1	N/A	1	1	N/A	N/A	1	0	N/A	0	N/A	N/A	N/A	**5**
	**Genital**	Haemospermia	1	0	0	1	0	0	1	N/A	0	N/A	N/A	0	1	N/A	N/A	0	0	N/A	0	N/A	N/A	N/A	**2**
		Dyspareunia	1	0	0	1	0	0	1	N/A	0	N/A	N/A	0	1	N/A	N/A	0	0	N/A	0	N/A	N/A	N/A	**3**
		Dysorgasmia	1	0	0	1	0	0	1	N/A	0	N/A	N/A	0	1	N/A	N/A	0	0	N/A	0	N/A	N/A	N/A	**4**
		Genital pain	1	0	0	1	0	0	1	N/A	0	N/A	N/A	0	1	N/A	N/A	0	0	N/A	0	N/A	N/A	N/A	**6**
		Genital sores	1	0	0	1	0	0	1	N/A	0	N/A	N/A	0	1	N/A	N/A	0	0	N/A	0	N/A	N/A	N/A	**4**
		**N sx experienced**	**0**	**8**	**8**	**4**	**14**	**11**	**8**	**0**	**14**	**8**	**0**	**1**	**3**	**0**	**0**	**7**	**0**	**0**	**3**	**0**	**0**	**0**	

Note: Within the table: red colour = symptom experienced, 1 = *Schistosoma* eggs in urine or semen, 0 = no *Schistosoma* eggs in urine or semen, N/A = urine or semen sample not available for analysis.

**Figure 2.**
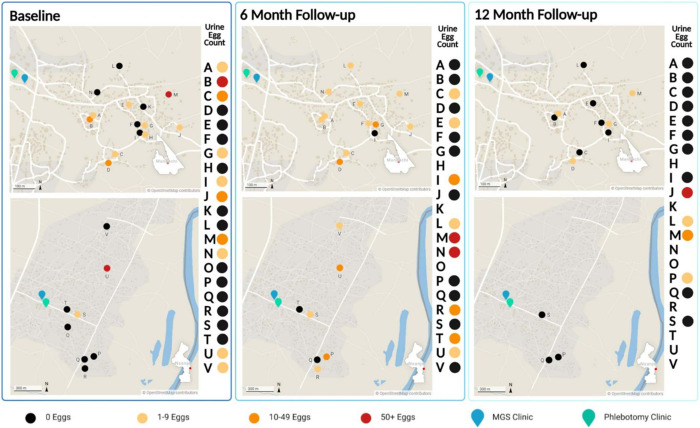
Map showing findings of semen microscopy of the study participants alongside those of urine filtration in the 2 communities around Samama school in Mangochi district and Mthawira school in Nsanje district of Southern Malawi where participants came from.

Five of the 10 participants who had *Schistosoma* eggs in their urine at baseline, remained egg-patent at 6-month follow-up, with 4 participants (E, L, R and T) who were urinary egg-patent for the first time, giving a prevalence of 47·4% (*n* = 19). At 12-month follow-up, 7 out of the 10 participants with *Schistosoma* eggs in their urine at baseline were present and participant J was urinary egg-patent at baseline and this time point while participant M was infected at all 3 time points. In total, 4 (J, L, M and P) had eggs in their urine (28·6%, *n* = 14) at this time point (Figure 2).

On semen microscopy, 10 out of 11 participants with *S. haematobium* eggs at baseline had persistent infection at 6-month follow-up, together with 6 participants who had eggs in their semen for the first time, showing a higher infection rate of 84·2% (*n* = 19). Two participants, G and M, also had *S. mattheei* eggs in semen.

Of the 13 participants who were available at 12-month follow-up, only 4 (A, D, G and M) had *S. haematobium* eggs in their semen which were persistent at all the time points, with 1 (participant G) having persistent *S. mattheei* eggs from 6-month follow-up, depicting a dual human and zoonotic infection at both time points. Two participants (A and D) had calcified *S. haematobium* eggs at this time point.

Only 1 of the 3 participants, D, who had changes in his semen observed at baseline, had normal features at subsequent follow-up time points. However, 4 additional participants (E, G, L and S) who also had *Schistosoma* eggs in their semen were observed to have abnormal changes ranging from loose semen consistency, few live or dead spermatozoa to azoospermia at 6- and 12-month time points.

Using real time PCR at 6-month follow-up, 14 of the 19 participants (73·7%) were positive for *Schistosoma* infection, with only 2 participants (E and R) who were infected for first time at this time point (Table 3 and Supplementary Table 1). Three participants had a possible hybrid infection at this time point. On 12-month follow-up, only 6 out of 13 participants had positive *Schistosoma* infection with no possible hybrid infection. All in all, only 6 of all recruited participants (B, C, F, L, P and S) had no positive *Schistosoma* infection on PCR at the end of the study.

**Table 3.** Results of molecular analysis on *Schistosoma* infection using real-time PCR of the participants’ semen

Participant ID	Age (years)	Baseline				6 months				12 months			
		Gen	Mito	HRM Tm1	HRM Tm2	Gen	Mito	HRM Tm1	HRM Tm2	Gen	Mito	HRM Tm1	HRM Tm2
A	19	*Sh*	*Smat*	*Smat*	0·0	*Sh*	0·0	*Smat*	0·0	*Sh*	*Smat*	*Smat*	0·0
B	18	*Sh*	*Sm*	*Sm*	0·0	*Shyp*	*Shyp*	*Shyp*	*Shyp*	0·0	0·0	0·0	0·0
C	18	*Sh*	*Smat*	*Smat*	0·0	*Sh*	0·0	0·0	0·0	0·0	0·0	0·0	0·0
D	18	*Sh*	0·0	0·0	0·0	*Sh*	*Sc*	*Sc*	0·0	*Sh*	0·0	0·0	0·0
E	21	0·0	0·0	0·0	0·0	*Sh*	*Smat*	*Smat*	0·0	*Sh*	0·0	0·0	0·0
F	28	*Shyp*	0·0	*Shyp*	*Sm*	*Sh*	*Smat*	*Smat*	0·0	0·0	0·0	0·0	0·0
G	27	*Sh*	*Smat*	*Smat*	0·0	*Sh*	*Smat*	*Smat*	0·0	*Sh*	*Smat*	*Smat*	0·0
H	19	*Sh*	0·0	0·0	0·0	N/A	N/A	N/A	N/A	N/A	N/A	N/A	N/A
I	30	*Sh*	0·0	0·0	0·0	*Sh*	0·0	0·0	0·0	*Sh*	0·0	0·0	0·0
J	22	*Sh*	0·0	0·0	0·0	*Sh*	*Sc*	*Sc*	0·0	N/A	N/A	N/A	N/A
K	18	0·0	0·0	0·0	0·0	N/A	N/A	N/A	N/A	N/A	N/A	N/A	N/A
L	21	*Sh*	0·0	0·0	0·0	0·0	0·0	0·0	0·0	0·0	0·0	0·0	0·0
M	18	*Sh*	0·0	0·0	0·0	*Shyp*	*Shyp*	*Shyp*	0·0	*Sh*	0·0	0·0	0·0
N	32	*Sh*	*Sm*	*Sm*	0·0	0·0	0·0	0·0	0·0	N/A	N/A	N/A	N/A
O	32	0·0	0·0	0·0	0·0	N/A	N/A	N/A	N/A	N/A	N/A	N/A	N/A
P	39	*Sh*	0·0	0·0	0·0	*Sh*	0·0	0·0	0·0	0·0	0·0	0·0	0·0
Q	30	0·0	0·0	0·0	0·0	0·0	0·0	0·0	0·0	0·0	0·0	0·0	0·0
R	26	0·0	0·0	0·0	0·0	*Shyp*	*Shyp*	*Shyp*	0·0	N/A	N/A	N/A	N/A
S	19	*Sh*	0·0	0·0	0·0	*Sh*	0·0	0·0	0·0	0·0	0·0	0·0	0·0
T	22	0·0	0·0	0·0	0·0	0·0	0·0	0·0	0·0	N/A	N/A	N/A	N/A
U	22	*Sh*	0·0	0·0	0·0	*Sh*	*Sc*	*Sc*	0·0	N/A	N/A	N/A	N/A
V	25	0·0	*Smat*	*Smat*	0·0	0·0	0·0	0·0	0·0	N/A	N/A	N/A	N/A

Note: Gen = generic, Mito = mitochondrial, HRM = high-resolution melt, Tm = melting temperature, N/A = not available; *Sh* = *Schistosoma haematobium, Sm* = *Schistosoma mansoni, Smat* = *Schistosoma mattheei, Sc* = *Schistosoma curassoni, Shyp* = *Schistosoma* hybrid, 0·0 = no *Schistosoma* detected; colours: dark blue = *Schistosoma haematobium*; plum = *Schistosoma mansoni*; dark green = *Schistosoma mattheei*; orange = *Schistosoma curassoni*; red = *Schistosoma* hybrid.

Of those participants who had *Schistosoma* eggs in semen during the study, 4 had none in urine (D, F, G and S). Furthermore, participants Q and T had no eggs in urine nor semen, also with negative PCR.

All the 12 (54·5%) participants who had detectable HPV serotypes 16 and 18 in semen, associated with penile and cervical carcinoma at baseline, had no HPV detected during follow-up at both time points (Table 4 and Supplementary Table 2). On STIs, particularly *T. vaginalis*, only one participant was positive at 6-month follow-up and none at 12-month follow-up, showing a downward trend of both infections.

**Table 4.** Results of the real time PCR for Human Papilloma Virus (HPV) serotypes and STIs – *Trichomonas vaginalis* in comparison with and *Schistosoma* species

Participant ID	Age (years)	Baseline					6 months					12 months				
		Gen	HRM Tm1	HRM Tm2	HPV type	STI *Tv*	Gen	HRM Tm1	HRM Tm2	HPV type	STI *Tv*	Gen	HRM Tm1	HRM Tm2	HPV type	STI *Tv*
A	19	*Sh*	*Smat*	*Smat*	16	0·0	*Sh*	*Smat*	0·0	–	0·0	*Sh*	*Smat*	*Smat*	–	0·0
B	18	*Sh*	*Sm*	*Sm*	Other	0·0	*Shyp*	*Shyp*	*Shyp*	–	0·0	0·0	0·0	0·0	–	0·0
C	18	*Sh*	*Smat*	*Smat*	Other	0·0	*Sh*	0·0	0·0	–	0·0	0·0	0·0	0·0	–	0·0
D	18	*Sh*	0·0	0·0	18	0·0	*Sh*	*Sc*	0·0	–	0·0	*Sh*	0·0	0·0	–	0·0
E	21	0·0	0·0	0·0	Other	0·0	*Sh*	*Smat*	0·0	–	0·0	*Sh*	0·0	0·0	–	0·0
F	28	*Shyp*	*Shyp*	*Sm*	16	0·0	*Sh*	*Smat*	0·0	–	0·0	0·0	0·0	0·0	–	0·0
G	27	*Sh*	*Smat*	0·0	18	0·0	*Sh*	*Smat*	0·0	–	0·0	*Sh*	*Smat*	*Smat*	–	0·0
H	19	*Sh*	0·0	0·0	Other	0·0	N/A	N/A	N/A	N/A	N/A	N/A	N/A	N/A	N/A	N/A
I	30	*Sh*	0·0	0·0	16	*Tv*	*Sh*	0·0	0·0	–	0·0	*Sh*	0·0	0·0	–	0·0
J	22	*Sh*	0·0	0·0	–	0·0	*Sh*	*Sc*	0·0	–	0·0	N/A	N/A	N/A	N/A	N/A
K	18	0·0	0·0	0·0	–	*Tv*	N/A	N/A	N/A	N/A	N/A	N/A	N/A	N/A	N/A	N/A
L	21	*Sh*	0·0	0·0	Other	*Tv*	0·0	0·0	0·0	–	0·0	0·0	0·0	0·0	–	0·0
M	18	*Sh*	0·0	0·0	–	*Tv*	*Shyp*	*Shyp*	0·0	–	0·0	*Sh*	0·0	0·0	–	0·0
N	32	*Sh*	*Sm*	0·0	16	*Tv*	0·0	0·0	0·0	–	0·0	N/A	N/A	N/A	N/A	N/A
O	32	0·0	0·0	0·0	–	0·0	N/A	N/A	N/A	–	0·0	N/A	N/A	N/A	N/A	N/A
P	39	*Sh*	0·0	0·0	Other	*Tv*	*Sh*	0·0	0·0	–	0·0	0·0	0·0	0·0	–	0·0
Q	30	0·0	0·0	0·0	–	0·0	0·0	0·0	0·0	–	0·0	0·0	0·0	0·0	–	0·0
R	26	0·0	0·0	0·0	–	0·0	*Shyp*	*Shyp*	0·0	–	*Tv*	N/A	N/A	N/A	N/A	N/A
S	19	*Sh*	0·0	0·0	–	0·0	*Sh*	0·0	0·0	–	0·0	0·0	0·0	0·0	–	0·0
T	22	0·0	0·0	0·0	–	0·0	0·0	0·0	0·0	–	0·0	N/A	N/A	N/A	N/A	N/A
U	22	*Sh*	0·0	0·0	–	0·0	*Sh*	*Sc*	0·0	–	0·0	N/A	N/A	N/A	N/A	N/A
V	25	0·0	*Smat*	0·0	–	0·0	0·0	0·0	0·0	–	0·0	N/A	N/A	N/A	N/A	N/A

Note: Gen = generic, HRM = high-resolution melt, Tm = melting temperature, *Tv* = *T. vaginalis*; N/A = not available, *Sh* = *Schistosoma haematobium, Sm* = *Schistosoma mansoni, Smat* = *Schistosoma mattheei, Sc* = *Schistosoma curassoni, Shyp* = *Schistosoma* hybrid, 0·0 = no *Schistosoma* detected; colours: dark blue = *Schistosoma haematobium*; plum = *Schistosoma mansoni*; dark green = *Schistosoma mattheei*; orange = *Schistosoma curassoni*; red = *Schistosoma* hybrid; yellow = HPV serotypes 16 or 18 or others; purple = *T. vaginalis.*

## Discussion

As described previously, here in these 2 southern communities of Malawi, MGS remains a seldomly recognised, undiagnosed condition in endemic areas, where it is as commonly prevalent as urogenital schistosomiasis, and with coinfection signatures with *S. mattheei* (Kayuni et al. 2024a). This study is a 12-month follow-up to our initial study comprising a cohort of 22 men where *Schistosoma* eggs was detected in semen of 11 men (50·0%) with 16 men (72·7%) positive on real-time PCR for *Schistosoma* infection, conducted at 2 time points of 6 months each. It was noted that despite taking a directly observed standard dose of praziquantel treatment, 40 mg/kg body weight, some participants had persistent or possible re-infections.

An important observation is that four participants had *Schistosoma* eggs only in semen during the study, which is consistent with similar studies, showing that the individuals can have MGS due to worms inhabiting the blood vessels around the genital organs, rather than contamination of the urethra from urinary schistosomiasis (Kayuni et al. 2019a, 2021; Bustinduy et al. 2022). Hence, the critical importance of analysing semen samples as a way of diagnosing MGS.

Previous reports on MGS associated with non-human schistosomes have been inadequate and mostly case reports of people visiting to endemic areas, despite studies done in Malawi and Madagascar describing significant burden of MGS (Leutscher et al. 2000; Kayuni et al. 2021, 2023a). Furthermore, the extent of the morbidity resulting from these zoonotic and hybrid MGS infections remains poorly understood although these schistosomes in SSA are increasingly becoming recognized as an emerging public health problem (Webster et al. 2019; Stothard et al. 2020) and future challenge in gaining adequate environmental transmission control (Stothard et al. 2024).

The proximity of most study participants to domesticated livestock such as goats, sheep and cattle in the study area which have shown to harbour both human and zoonotic mixed infections (Juhász et al. 2024a, 2024b) could have significantly contributed to the level of MGS. Certainly, some participants were observed to have hybrid infections during the follow-up using our novel two-tube real-time PCR (Cunningham et al. 2024), which brings about the need for advanced molecular diagnostics to improve detection of such diseases. Furthermore, one health initiatives can aid in addressing the emerging zoonotic and hybrid infections which is being worsened with adverse climate change, thereby affecting efforts in the control and elimination of schistosomiasis as public health problem in endemic areas (Mbabazi et al. 2011; Shukla et al. 2023).

Although some participants had changes in their semen, namely haemospermia, changes in semen colour and consistency and azoospermia which have also been reported in other studies, only one had resolved these abnormalities during follow-up. This highlights the need for earlier treatment with praziquantel to experience its beneficial effects, apart from other interventions to prevent such infections as well as improving diagnostic services, healthcare professional awareness and capacity in disease management (Mbabazi et al. 2011; Shukla et al. 2023).

Certainly, most health facilities in the endemic areas use the syndromic approach in the STI management (WHO 2005, 2021). These genital symptoms utilized are similar to those attributed to MGS, also observed in our study, which can result in under-diagnosis and inappropriate management. Also, MGS has been observed to play a role in the progression of other infections such as human immunodeficiency virus (HIV) among other factors, as previously observed in increasing seminal viral load of dually infected men (Midzi et al. 2017; Kayuni et al. 2023b). Therefore, there is need for more advocacy for appropriate diagnostic and management resources together with awareness and capacity building among community people and healthcare providers in schistosomiasis-endemic areas.

As earlier alluded to, the study was limited in the low number of study participants owing to the sensitivity of the condition which calls for larger stratified studies in other endemic areas to provide a representative picture of the MGS outcome.

## Conclusion

Human, zoonotic and hybrid schistosomes continues to being incriminated in causing MGS significantly, hence posing a One Health challenge in management and control measures in resource poor settings. This calls for more tailored public health education campaigns in the endemic communities, awareness of MGS among primary healthcare workers and local animal health experts and collaborations among these stakeholders and National Schistosomiasis Control Programme in handling this burden and address it adequately.

## Supporting information

Mainga et al. supplementary material Mainga et al. supplementary material

## Data Availability

The datasets generated and analysed for this study has been included in this manuscript.

## Supplementary material

The supplementary material for this article can be found at https://doi.org/10.1017/S0031182025100942.
