# Supplementary material for: A longitudinal study of men with male genital schistosomiasis in southern Malawi associated with human, zoonotic and hybrid schistosomes
Source: Parasitology. 2025 Oct 7;152(14):1446–58. doi: 10.1017/S0031182025100942 (PMC13124307; doi:10.1017/S0031182025100942)
Supplement: Mainga et al. supplementary material [file S0031182025100942sup001.docx]

**Supplementary Table S1: Detailed results of molecular analysis on *Schistosoma* infection using real-time PCR of the participants’ semen.**

| **Participant ID** | **Age (years)** | **Baseline** | | | | **6-months** | | | | **12-months** | | | |
| --- | --- | --- | --- | --- | --- | --- | --- | --- | --- | --- | --- | --- | --- |
|  |  | **Cq Gen** | **Cq Mito** | **HRM Tm1(°c)** | **HRM Tm2(°c)** | **Cq Gen** | **Cq Mito** | **HRM Tm1(°c)** | **HRM Tm2(°c)** | **Cq Gen** | **Cq Mito** | **HRM Tm1(°c)** | **HRM Tm2(°c)** |
| A | 19 | 16.8 | 27.3 | 71.2 | 0.0 | 28.5 | 0.0 | 71.9 | 0.0 | 23.5 | 29.0 | 71.6 | 0.0 |
| B | 18 | 18.5 | 28.9 | 76.3 | 0.0 | 31.7 | 32.1 | 71.9 | 76.3 | 0.0 | 0.0 | 0.0 | 0.0 |
| C | 18 | 16.5 | 31.2 | 71.5 | 0.0 | 30.8 | 0.0 | 0.0 | 0.0 | 0.0 | 0.0 | 0.0 | 0.0 |
| D | 18 | 20.4 | 0.0 | 0.0 | 0.0 | 28.5 | 32.4 | 72.1 | 0.0 | 20.4 | 0.0 | 0.0 | 0.0 |
| E | 21 | 0.0 | 0.0 | 0.0 | 0.0 | 27.7 | 33.0 | 71.9 | 0.0 | 28.7 | 0.0 | 0.0 | 0.0 |
| F | 28 | 24.1 | 0.0 | 71.4 | 76.2 | 28.9 | 26.8 | 71.4 | 0.0 | 0.0 | 0.0 | 0.0 | 0.0 |
| G | 27 | 22.4 | 28.7 | 71.4 | 0.0 | 25.0 | 24.8 | 71.6 | 0.0 | 22.5 | 28.9 | 71.7 | 0.0 |
| H | 19 | 25.3 | 0.0 | 0.0 | 0.0 | N/A | N/A | N/A | N/A | N/A | N/A | N/A | N/A |
| I | 30 | 23.6 | 0.0 | 0.0 | 0.0 | 31.4 | 0.0 | 0.0 | 0.0 | 34.9 | 0.0 | 0.0 | 0.0 |
| J | 22 | 25.5 | 0.0 | 0.0 | 0.0 | 34.4 | 25.1 | 72.3 | 0.0 | N/A | N/A | N/A | N/A |
| K | 18 | 0.0 | 0.0 | 0.0 | 0.0 | N/A | N/A | N/A | N/A | N/A | N/A | N/A | N/A |
| L | 21 | 37.8 | 0.0 | 0.0 | 0.0 | 0.0 | 0.0 | 0.0 | 0.0 | 0.0 | 0.0 | 0.0 | 0.0 |
| M | 18 | 20.4 | 0.0 | 0.0 | 0.0 | 23.0 | 29.0 | 74.2 | 0.0 | 30.6 | 0.0 | 0.0 | 0.0 |
| N | 32 | 23.7 | 25.4 | 76.1 | 0.0 | 0.0 | 0.0 | 0.0 | 0.0 | N/A | N/A | N/A | N/A |
| O | 32 | 0.0 | 0.0 | 0.0 | 0.0 | N/A | N/A | N/A | N/A | N/A | N/A | N/A | N/A |
| P | 39 | 36.6 | 0.0 | 0.0 | 0.0 | 27.4 | 0.0 | 0.0 | 0.0 | 0.0 | 0.0 | 0.0 | 0.0 |
| Q | 30 | 0.0 | 0.0 | 0.0 | 0.0 | 0.0 | 0.0 | 0.0 | 0.0 | 0.0 | 0.0 | 0.0 | 0.0 |
| R | 26 | 0.0 | 0.0 | 0.0 | 0.0 | 24.2 | 34.1 | 71.9 | 0.0 | N/A | N/A | N/A | N/A |
| S | 19 | 28.7 | 0.0 | 0.0 | 0.0 | 29.0 | 0.0 | 0.0 | 0.0 | 0.0 | 0.0 | 0.0 | 0.0 |
| T | 22 | 0.0 | 0.0 | 0.0 | 0.0 | 0.0 | 0.0 | 0.0 | 0.0 | N/A | N/A | N/A | N/A |
| U | 22 | 21.6 | 0.0 | 0.0 | 0.0 | 24.1 | 32.9 | 72.2 | 0.0 | N/A | N/A | N/A | N/A |
| V | 25 | 0.0 | 32.3 | 71.9 | 0.0 | 0.0 | 0.0 | 0.0 | 0.0 | N/A | N/A | N/A | N/A |

Note: Cq = Threshold cycle, Gen = Generic, Mito = Mitochondrial, HRM = High-Resolution Melt, Tm = melting temperature, C = degrees Celsius, N/A = Not Available; Colours: Dark Blue = *Schistosoma haematobium*; Plum = *Schistosoma mansoni*; Dark Green = *Schistosoma mattheei*; Orange = *Schistosoma curassoni*; Red = *Schistosoma* hybrid

**Supplementary Table S2: Detailed results of the real time PCR for Human Papilloma Virus (HPV) serotypes and STIs - *Trichomonas vaginalis* in comparison with and *Schistosoma* species**

| **Participant ID** | **Age (years)** | **Baseline** | | | | | **6-months** | | | | | **12-months** | | | | |
| --- | --- | --- | --- | --- | --- | --- | --- | --- | --- | --- | --- | --- | --- | --- | --- | --- |
|  |  | **Cq Gen** | **HRM Tm1(°c)** | **HRM Tm2(°c)** | **HPV type** | **STI**  ***Tv*** | **Cq Gen** | **HRM Tm1(°c)** | **HRM Tm2(°c)** | **HPV type** | **STI**  ***Tv*** | **Cq Gen** | **HRM Tm1(°c)** | **HRM Tm2(°c)** | **HPV type** | **STI**  ***Tv*** |
| A | 19 | 16.8 | 71.2 | 0.0 | 16 | 0.0 | 28.5 | 71.9 | 0.0 | - | 0.0 | 23.5 | 71.6 | 0.0 | - | 0.0 |
| B | 18 | 18.5 | 76.3 | 0.0 | Other | 0.0 | 31.7 | 71.9 | 76.3 | - | 0.0 | 0.0 | 0.0 | 0.0 | - | 0.0 |
| C | 18 | 16.5 | 71.5 | 0.0 | Other | 0.0 | 30.8 | 0.0 | 0.0 | - | 0.0 | 0.0 | 0.0 | 0.0 | - | 0.0 |
| D | 18 | 20.4 | 0.0 | 0.0 | 18 | 0.0 | 28.5 | 72.1 | 0.0 | - | 0.0 | 20.4 | 0.0 | 0.0 | - | 0.0 |
| E | 21 | 0.0 | 0.0 | 0.0 | Other | 0.0 | 27.7 | 71.9 | 0.0 | - | 0.0 | 28.7 | 0.0 | 0.0 | - | 0.0 |
| F | 28 | 24.1 | 71.4 | 76.2 | 16 | 0.0 | 28.9 | 71.4 | 0.0 | - | 0.0 | 0.0 | 0.0 | 0.0 | - | 0.0 |
| G | 27 | 22.4 | 71.4 | 0.0 | 18 | 0.0 | 25.0 | 71.6 | 0.0 | - | 0.0 | 22.5 | 71.7 | 0.0 | - | 0.0 |
| H | 19 | 25.3 | 0.0 | 0.0 | Other | 0.0 | N/A | N/A | N/A | N/A | N/A | N/A | N/A | N/A | N/A | N/A |
| I | 30 | 23.6 | 0.0 | 0.0 | 16 | 29.3 | 31.4 | 0.0 | 0.0 | - | 0.0 | 34.9 | 0.0 | 0.0 | - | 0.0 |
| J | 22 | 25.5 | 0.0 | 0.0 | - | 0.0 | 34.4 | 72.3 | 0.0 | - | 0.0 | N/A | N/A | N/A | N/A | N/A |
| K | 18 | 0.0 | 0.0 | 0.0 | - | 34.1 | N/A | N/A | N/A | N/A | N/A | N/A | N/A | N/A | N/A | N/A |
| L | 21 | 37.8 | 0.0 | 0.0 | Other | 34.5 | 0.0 | 0.0 | 0.0 | - | 0.0 | 0.0 | 0.0 | 0.0 | - | 0.0 |
| M | 18 | 20.4 | 0.0 | 0.0 | - | 35.1 | 23.0 | 74.2 | 0.0 | - | 0.0 | 30.6 | 0.0 | 0.0 | - | 0.0 |
| N | 32 | 23.7 | 76.1 | 0.0 | 16 | 23.3 | 0.0 | 0.0 | 0.0 | - | 0.0 | N/A | N/A | N/A | N/A | N/A |
| O | 32 | 0.0 | 0.0 | 0.0 | - | 0.0 | N/A | N/A | N/A | - | 0.0 | N/A | N/A | N/A | N/A | N/A |
| P | 39 | 36.6 | 0.0 | 0.0 | Other | 37.5 | 27.4 | 0.0 | 0.0 | - | 0.0 | 0.0 | 0.0 | 0.0 | - | 0.0 |
| Q | 30 | 0.0 | 0.0 | 0.0 | - | 0.0 | 0.0 | 0.0 | 0.0 | - | 0.0 | 0.0 | 0.0 | 0.0 | - | 0.0 |
| R | 26 | 0.0 | 0.0 | 0.0 | - | 0.0 | 24.2 | 71.9 | 0.0 | - | 26.6 | N/A | N/A | N/A | N/A | N/A |
| S | 19 | 28.7 | 0.0 | 0.0 | - | 0.0 | 29.0 | 0.0 | 0.0 | - | 0.0 | 0.0 | 0.0 | 0.0 | - | 0.0 |
| T | 22 | 0.0 | 0.0 | 0.0 | - | 0.0 | 0.0 | 0.0 | 0.0 | - | 0.0 | N/A | N/A | N/A | N/A | N/A |
| U | 22 | 21.6 | 0.0 | 0.0 | - | 0.0 | 24.1 | 72.2 | 0.0 | - | 0.0 | N/A | N/A | N/A | N/A | N/A |
| V | 25 | 0.0 | 71.9 | 0.0 | - | 0.0 | 0.0 | 0.0 | 0.0 | - | 0.0 | N/A | N/A | N/A | N/A | N/A |

Note: Cq = Threshold cycle, Gen = Generic, HRM = High-Resolution Melt, Tm = melting temperature, C = degrees Celsius, N/A = Not Available; Colours: Dark Blue = *Schistosoma haematobium*; Plum = *Schistosoma mansoni*; Dark Green = *Schistosoma mattheei*; Orange = *Schistosoma curassoni*; Red = *Schistosoma* hybrid; Yellow = HPV serotypes 16 or 18; Purple = *T. vaginalis*
